# The Pathology and Treatment of Leucorrhœa

**Published:** 1855-09

**Authors:** W. Tyler Smith

**Affiliations:** Member of the Royal College of Physicians, Physician Accoucheur to St. Mary’s Hospital; Lecturer on Midwifery and the Diseases of Women in St. Mary’s Hospital Medical School; Vice-President of the Medical Society of London; Honorary Fellow of the Obstetrical Society of Dublin, etc., etc. Philadelphia, 1855: Blanchard & Lea.


*The Pathology and Treatment of Leucorrhœa.* By W. Tyler Smith, M. D., Member of the Royal College of Physicians, Physician Accoucheur to St. Mary's Hospital; Lecturer on Midwifery and the Diseases of Women in St. Mary's Hospital Medical School; Vice-President of the Medical Society of London; Honorary Fellow of the Obstetrical Society of Dublin, etc., etc. Philadelphia, 1855: Blanchard & Lea.

There is great truth in the remarks with which Dr. Smith commences the present treatise, which is to be viewed as the more full development of the leading points in reference to leucorrhœa and its allied disorders, advanced in a memoir presented by the author to the Royal Medical and Chirurgical Society of London, and published in the thirty-fifth volume of its *Transactions.*

"Few topics," he very justly observes, "have been more discussed during recent years than those relating to the pathology and treatment of disorders of the uterine organs attended by discharges. But it must be confessed that discussion has expended itself chiefly upon verbal criticism, and contributed little towards the more clear comprehension of this department of medicine; there has been much of argument, but, as I submit, little of rigorous examination. Tongue and pen have been plied with remarkable assiduity, yet the difficulties surrounding the subject have been rather increased than diminished. On many points of diagnosis and pathology, apparently the most easy of solution, the greatest uncertainty still prevails. This uncertainty naturally extends itself to the subject of treatment, and shows itself at every turn in practice. The various lessons, real and supposed, of the os and cervix uteri, ulceration, induration, and inflammation, have been attacked or defended by their partisans and opponents with the hottest zeal. In the diagnosis of these affections, some have practised instrumental examinations to an extent hitherto unprecedented in this country, while others have condemned such examinations altogether. As regards treatment, we see at one time injections, at another pessaries, at another cauterizations, are assailed with the utmost vigor, leaving the conscientious practitioner bewildered and uncertain as to what are really the best methods of controlling the confessedly troublesome and prevalent maladies for which these and other appliances are in turn vaunted or anathematized."

To acquire, if possible, more correct and definite views in regard to the pathology and proper treatment of a class of diseases, of the character and management of which, though much has been said and written, little is actually known, Dr. Smith, aided by the microscope, has investigated anew the nature and structure of the several tissues of the vagina and os and cervix uteri, the secretions of these parts in the healthy state, and their several morbid conditions, from that attended by a simple augmentation of the normal secretions, onward, through the several stages of abrasion and simple ulceration of the vagina, and of inflammation, abrasion, ulceration, induration, and hypertrophy of the mouth and neck of the uterus.

This investigation is one replete with interest, and has been conducted in a systematic and skilful manner by Dr. Smith. Although the correctness of some of the positions laid down by him may admit of dispute, he has nevertheless succeeded in furnishing the foundations for a more correct pathology of leucorrhœa than has hitherto existed, and for a more rational and successful treatment of the several morbid conditions of the uterus and vagina, in connection with which leucorrhœal discharges are liable to occur.

"If as I have previously shown," says Dr. S., "great discrepancy of opinion has prevailed respecting the sources of the healthy secretions of the vagina and canal of the cervix uteri, there has been still greater confusion as regards the seat and constitution of the morbid utero-vaginal discharges. No one had inquired minutely into the nature of these discharges, so that current opinions upon the subject have had no better foundation than guessing and hypothesis. Let any one who doubts the correctness of what is here advanced, examine for himself the doctrines hitherto taught respecting leucorrhœa, and he will find that some refer to the vulvo-vaginal glands as the chief seat of leucorrhœal discharges; that others refer to the vagina as a great follicular tract from which the principal amount of these discharges proceeds; that others, again, look to the cavity of the fundus uteri and its mucous lining as the great source of uterine mucous secretions. As regards the causes of these discharges, some have limited their attention to the sexual organs, while others have looked to the conditions of remote parts of the body, for the explanation of leucorrhœal disorders. It would, indeed, be easy to fill a volume with the discordant accounts which, in the absence of a knowledge of the minute anatomy of the parts involved, have been given of the nature and source of leucorrhœal discharges. One or two authors only have referred to the canal of the cervix uteri as the principal seat of mischief in leucorrhœa; but their teachings have been uncertain and without proof, since no one, so far as I am aware, ever made a positive and minute examination into the subject, or recognized to the full extent the glandular organization of the cervical canal. No pathologist has hitherto formed anything like a just appreciation of the parts borne respectively by the vagina and the os and cervix uteri in the production of leucorrhœal discharges. Effects have been constantly mistaken for causes, and secondary phenomena have received the importance due to those which are primary, while in practice the most important structures have frequently escaped attention altogether. The consequence has been that some have recommended the most violent measures of treatment, while others have rejected all remedial measures except the most simple and inert. Meanwhile, this department of medicine has witnessed a contest which for virulence and acrimony has seldom been equalled in the profession."

According to the researches of Dr. S., the mucus secreted by the glands of the ostium vaginæ, in the absence of excitement, is so inconsiderable, or so mixed up with the scaly epithelium of the mucous surface of this part, that it is extremely difficult to ascertain precisely its microscopical qualities. In some women a profuse emission of fluid appears to take place from these glands during sexual intercourse. Like the other vaginal secretions, this mucus has an acid reaction.

"The mucus of the vaginal canal is not found in any considerable quantity in the healthy subject; it is only secreted in sufficient quantity to keep the mucous surface in a state of lubrication. It lies upon the mucous membrane as a milky fluid, containing small curdy points or masses, and consists of a transparent or semi-transparent plasma, containing an abundance of scaly epithelium and its debris. The plasma of the vaginal mucus appears, when first secreted, to resemble the plasma of the cervical mucus; but it is less viscid and tenacious. It is only after it has lain a short time upon the vaginal surface that it becomes curdled. The vaginal mucus is, as M. Donne first remarked, distinctly acid, and it is to the effect of the acid in coagulating the albumen of the mucus, and not to the presence of epithelium, that its curdled appearance is attributable."

"As regards secretion, the vagina is always pretty much in the same condition, except that the acidity is constantly increased during pregnancy; but the cervix uteri has to pass through various physiological changes during the performance of the functions of menstruation, pregnancy, parturition, and lactation. It becomes necessary, therefore, to consider the secretion of the canal of the cervix in these several states.

"In the *unimpregnated condition*, when the cervix uteri is found perfectly healthy, little or no discharge is seen issuing from the cervical cavity; but when the labia uteri are separated, the canal of the cervix appears to be full of its peculiar secretion. In examinations after death, in cases in which the uterine organs are in a healthy condition, the mucous crypts and the canal of the cervix are generally found filled with a clear, viscid mucus, so as to entirely block up the passage from the vagina to the cavity of the fundus. This appears to be the normal condition of the cervical canal in the unimpregnated state. At each catamenial period the whole of the tenacious plug of mucus must be washed away by the menstrual fluid, as the latter may be seen escaping freely from the os uteri at these times; but, in a few days after the completion of the period, the mucous plug is again formed. When first secreted, the cervical mucus is less thick and viscid than it afterwards becomes. Thus, it would seem to be the function of the glandular structure of the cervix, in the unimpregnated uterus, to secrete each month a sufficient quantity of viscid mucus to fill the canal of the cervix, the mucous follicles becoming comparatively inactive when this has been accomplished, until after its removal at the next flow of the catamenia. The function of the cervix is, therefore, in a certain sense, like that of the fundus, periodical; and we shall see hereafter that this periodicity is discernible in the diseased conditions of the cervix and its secretions. In healthy subjects the canal of the cervix is always full in the intervals between the menstrual periods, though there certainly seems nothing like a constant flow of the cervical mucus into the vagina. Just enough is secreted to fill the canal. The mucus itself consists of myriads of mucus corpuscles entangled in a transparent viscid plasma. The plasma is so tenacious that the mucus-corpuscles are found to be arranged in strings when placed under the microscope, and individual corpuscles are frequently seen to be elongated from the same cause.

"The use of the cervical mucus is probably two-fold. In the first place, it closes the cervix uteri, and defends the cavity of the fundus from external agencies as completely as though it were a shut sac. In the second place, it appears to afford a suitable medium for the passage of the spermatozoa through the cervix uteri into the uterine cavity."

"After the commencement of *pregnancy*, the periodical functions of the uterus cease, and in the generality of cases the plug of viscid mucus, when it is once formed, continues for the most unremoved until the commencement of labor." "Generally, during gestation, the lowest part of the plug is to a slight extent constantly wearing away, and is discharged in the form of debris into the vagina; but the secretion from the cervix goes only to such an extent as to keep the os and cervix closed. In other cases, the secretion is more profuse, but the cervix is kept full by an increased secretion from the glandular structures. The mucus plug formed during pregnancy is firmer than the mucus filling the cervix in the intervals between the monthly periods in the unimpregnated state, particularly at its lowest part, where it is perfectly white and opaque. In the upper parts of the cervix it is clear and transparent. The plug consists, in the upper part of the cervix, entirely of mucus globules and plasma; but in the lower portions of the plug these elements are mized with scaly epithelium in considerable quantity."

During *parturition*, the canal of the cervix secretes a quantity of mucus having a more fluid character than the plug of pregnancy. This secretion continues throughout the act of partnrition. The os uteri and vagina are freely lubricated by it.

"It has been generally considered," remarks Dr. S., "a vaginal secretion, partly from the fact of its being found upon the vaginal surface, and partly because no minute inquiry into its nature has ever been made. There is, however, no evidence that the vagina secretes much more profusely during labor than at any other time, and there could hardly be a profuse secretion from the vaginal mucus surface without such a shedding of epithelium as would leave the subjacent structures irritable and painful. Microscopical examination proves that the mucus found in the vagina is chiefly the product of the glands of the cervical canal. At the commencement of labor the discharge is white and opaque; but as labor proceeds, and after the plug of pregnancy has escaped, it becomes clear and transparent. It is now of the consistence of white of egg, alkaline in character, and consists almost entirely of tenacious plasma and an immense quantity of mucus globules, intermixed with scaly epithelium."

"Upon the completion of natural labor, these glands, those of the cervix uteri, continue to secrete with considerable activity, and their secretion forms a part of the lochial discharge. In many cases the last secretion which appears after the cessation of the lochia, is the viscid secretion of the canal of the cervix. Thus it is, perhaps, during parturition that the glandular function of the canal of the cervix uteri is most actively performed. The glandular element seems of more importance, at this time, than either in the unimpregnated state of the uterus or during the course of pregnancy. The uses of the secretion in lubricating the os and cervix uteri and the vagina during labor, are sufficiently obvious. The physiological condition which obtains at this time is also very closely allied to the pathological conditions which are present in the most common forms of leucorrhœa.

"Mild leucorrhœal discharge is very common during the *period of suckling*, particularly in women who do not menstruate. The secretion takes place, I have no doubt, chiefly from the glands of the cervical canal. In some cases, it is constant; in others it occurs only at the monthly periods. It is a common observation that, after labor, the application of the child to the breast causes after pains, and an increase of the lochial discharge. Uterine contraction and uterine pain are caused, for several days after delivery, every time the child is put to the breast, or the sensation of the draught is experienced. But it occasionally happens that this intimate relation between the breasts and the uterus is preserved to some extent during the whole of lactation, and I have met with some cases in which cervical leucorrhœal discharge constantly occurred whenever the child sucked the breast. Thus there is a marked tendency to increased secretion from the glands of the cervical canal during lactation. Sometimes the foundation of chronic leucorrhœa is laid at this time; but the increased mucus secretion generally ceases after weaning, and the re-establishment of menstruation. In women who are drained largely by leucorrhœal discharges while nursing, it is only to direct them to wean the child, and the discharge speedily diminishes."

We have given the foregoing general description of the normal secretions of the vagina and neck of the uterus, as laid down by Dr. S., because the views of that gentleman in relation to the pathology of leucorrhœa could not be well understood without it. His account of the secretions differs, in many particulars, from that commonly received; it would appear, however, to be based upon a series of accurate observations, and is, in all probability, correct.

Dr. S. divides leucorrhœa into two leading varieties, *cervical* or *mucus* leucorrhœa, and *vaginal* or *epithelial* leucorrhœa.

"It may be well," he observes, "to revert for a moment to the special differences which exist between the vagina and the cervical canal. The lining membrane of the vagina approaches in organization to the skin; it is covered by a thick layer of scaly epithelium; it contains in the greater part of its surface few, if any mucus follicles or glands; its secretion is acid, consisting entirely of plasma and epithelium, and the chief object of the secretion is the lubrication of the surface upon which it is formed. On the other hand, the lining of the canal of the cervix is a true mucus membrane; it is covered, in great part, by cylinder epithelium; it abounds with immense numbers of mucus follicles having a special arrangement; it pours forth a true mucus secretion, alkaline in character, and consisting of mucus corpuscles and plasma, with little or no epithelium; and this secretion has special uses to perform in the unimpregnated state, and in pregnancy and parturition. Leucorrhœa admits of a similar division. The first, and the most frequent and important is the mucus variety, consisting chiefly of mucus corpuscles and plasma, and secreated chiefly by the follicular canal of the cervix. The second is the epithelial variety, in which the discharge is vaginal, or is secreted by the vaginal portion of the os and cervix, and consists for the most part of scaly epithelium and its debris. These two varieties may, of course, exist in various degrees of combination; sometimes the one and sometimes the other preponderates, or is the original affection; but the chief importance must be given to cervical or mucus leucorrhœa, as being the most obstinate and common."

Mucous leucorrhœa, when simple and uncomplicated, is the result of a morbid activity of the glandular cervix. The discharge is at first nothing more than an unusual amount of the elements found in the healthy mucus of the cervical canal. In very severe cases, the mucus of the cervix becomes mixed with pus corpuscles, and the discharge is rendered muco-purulent in character, or the surface of the canal and the os uteri becomes so irritable as to bleed on the slightest irritation, blood corpuscles being added as another element of the discharge. When the quantity of blood is large and speedily evacuated from the vagina, the discharge resembles the menstrual flux in color; but when small in quantity and evacuated slowly, it gives to the discharge a greenish or brownish tint. When in cases of simple leucorrhœa the discharge is profuse and long continued, it often proves a serious drain to the constitution, and gives rise to functional or more serious disorders in different parts of the body. In other cases the secretion is so profuse and watery that the traces of viscidity are nearly lost. This excessive watery secretion, when long continued, is a source not only of inconvenience, but of great debility. Patients suffering from cervical leucorrhœa, besides becoming extremely debilitated by the amount of the discharge, may, also, become hectic from purulent secretion and absorption, or they may be rendered anæmic by the sanguineous complication. In the worst cases, the discharges, in their physical appearances, may resemble those in carcinoma.

"The discharge in *vaginal leucorrhœa* may arise, chiefly, either from the lower portion of the vaginal membrane, or from that part which is reflected over the cervix; but in severe cases, the whole surface of the vagina is involved. The secretion in these cases generally consists entirely of epithelium, in every possible phase of development, mixed with acid mucus plasma." "If the case be acute, there are no old and broken scales, such as are found in the healthy secretion, the epithelium being separated too rapidly, in the formation and flow of the discharge, to admit of their coming to maturity and wearing away in the vagina. In mild cases, when the separation is more slow, ripe and well worn, scales are sometimes present. When the vaginal form of leucorrhœa becomes very severe, the villi become affected, and not only is epithelium separated with extraordinary rapidity, but pus is formed upon the irritable sub-epithelial or villous surface, which, when mixed with the epithelial matter, can hardly be distinguished from the mucus corpuscles of the cevix, mixed with scaly epithelium. The state of the vagina, as seen by the eye, will, however, remove all doubt as to the nature of the discharge in these cases. A further complication of vaginal leucorrhœa may occur, as when portions of the vaginal surface are so abraded that blood globules escape and are mixed with the other constituents of the vaginal discharge. The vaginal secretions now described are those most commonly found in vaginal or epithelial leucorrhœa; but there is another form of vaginal discharge which deserves consideration." In this, "the epithelium is thrown off in large shreds or pieces, while the pavement-like arrangement of the scales is perfectly preserved. The laminæ frequently have upon them marks of the rugæ of the vagina, and somewhat resemble the cuticle in cases of acute desquamation of the surface of the body. The under surfaces of these masses are also rough from the indentations of the vaginal papillæ. Sometimes, on making a specular examination in these cases, the whole surface of the vagina is seen covered with a white coating, which may be removed by a forceps in membranous pieces of considerable extent and thickness. This affection may be attended with a slight discharge from the sub-epithelial surface; but in many cases the vagina does contain more secretion than usual, or it may be unnaturally dry. In all epithelial affections of the vagina the discharge is acid; but the acidity is particularly marked in this—the membranous form of leucorrhœa, as it may be termed. Some of the instances in which I have seen this affection in its most marked form, have been in cases of pregnancy. I have sometimes had patients to bring me a mass as large as a walnut, consisting of pieces of the epithelial coat of the vagina rolled up like paper; or I have seen a tumblerful of water rendered perfectly thick with the quantity of shreds removed from the vagina by a single injection.

"In these cases, the simple shedding of the epithelium in great abundance, and the desquamation of the epithelium in masses, might be called *Epithelial Vaginitis*, while the purulent form of the disorder, in which the villi are affected, might be called *Villous Vaginitis*."

So much for the simple forms of leucorrhœa; its sequelæ, when allowed to continue unchecked, are, according to Dr. S., inflammation, abrasion, ulceration, and hypertrophy of the os and cervix uteri, and abrasion and superficial ulceration of the vagina. It is the conviction of Dr. S., that, in the majority of those diseased conditions of the os and cervix uteri, which have of late been assigned so prominent a rank as distinct and independent affections in medical discussions, are in the majority of cases secondary affections, cervical leucorrhœa being, in fact, the primary and most essential disease. In maintaining the important part played by the cervical secretions in inducing morbid conditions of the os uteri, he does not wish, however, to be understood as saying that they are the only causes of these conditions. But even when disease of the os and cervix uteri has been induced by other causes, cervical leucorrhœa is almost invariably produced, and it generally tends to aggravate the diseased conditions of those parts.

Each of the conditions referred to by the author as sequelæ of leucorrhœa are separately considered. The extent to which we have already been led in our quotations from the work before us, admonishes us of the necessity of passing over the interesting remarks presented in reference to these sequelæ.

The constitutional and other derangements consequent upon protracted leucorrhœa come next under consideration. They are general debility, stomachic derangement, amenorrheœa or uterine hemorrhage, anæmia, hectic fever, derangements of the nervous system, pains in various parts of the body, neuralgic affections of the neck of the uterus, and troublesome affections of the bladder and rectum.

The diagnosis between leucorrhœa and cancer uteri, the relations between secondary syphilis and leucorrhæa, are then separately discussed, and a most interesting chapter is given on the relations of vaginal leucorrhœa to gonorrhœa in the female, uerethritis in the male, and the ophthalmia of new-born infants.

Dr. S. believes gonorrhœa in the female to be closely allied to vaginal leucorrhœa, and hence impure connection may be ranked as one of the causes of the latter. He has no doubt, also, that urethritis and inflammation of the glans penis may be induced in the male by a female laboring under spontaneous leucorrhœa arising independently of sexual intercourse, and that ophthalmia neonatorum may be caused in children born of females laboring under non-gonorrhœal leucorrhœa.

In chapter IX the author discusses the relations of leucorrhœa to disorders of the function of menstruation. He remarks that it is very rarely that leucorrhœa, with diseased conditions of the lower segment of the uterus, exists for any length of time without inducing some disorder of the catamenial function. This occurs chiefly in the cervical forms of leucorrhœa, or those cases of vaginal leucorrhæa in which the affection is confined to the surface of the os uteri and vaginal portion of the cervix. In some cases the leucorrhœal affection is the secondary disease, amenorrhœa, menorrhagia, or dysmenorrhœa having preceded it; but most commonly, according to Dr. S., the former is found to be the primary disorder in these cases, and the catamenial derangement has slowly followed upon chronic leucorrhœa.

Periodical leucorrhœa, or leucorrhœa vicarious to menstruation, receives a passing notice, and in the ensuing chapter (X.) the relations of leucorrhœa to sterility and abortion are very fully considered, which closes the discussion of the pathological character and relations of the disease.

As constitutional local causes of the disease, Dr. S. enumerates plethora, debility prolonged, the strumous habit, skin, diseases, climate, rectal, vesical, urethral, vaginal, and uterine irritation, gestation, abortion and labor. Appended to the chapter are some remarks on leucorrhœa in children.

At the risk of being accused of extending this notice to an unwarrantable length, we cannot refrain from presenting to our readers the concluding paragraph of this chapter of the work. It presents a general summary of the author's views in regard to the nature of leucorrhœa, and an expression of his opinion on a question of uterine pathology warmly discussed at the present period.

"From the whole tenor of the present work," says Dr. S., "it will be seen that I differ very strongly from the opinions which refer almost all the conditions upon which leucorrhœa depends to inflammation of the os and cervix uteri. I believe it cannot now be disputed that many of the affections of the os and cervix recently stated to constitute ulceration of the surface, are, in reality, only epithelial abrasions of more or less completeness. As regards ulceration, I believe the more searching examinations to which its asserted frequency has led, prove that its importance and frequency are much less than were formerly asserted. A modified view of the lesions supposed to constitute ulceration of the os and cervix uteri must certainly be taken; and, in a former chapter, I have stated the grounds upon which I believe that abrasions and superficial ulcerations of the os and cervix uteri, when they occur, are very frequently secondary affections, instead of primary disorders. In like manner, I

believe the vaunted importance of inflammation, as the great cause of uterine disorder, must be altogether modified. I think the term 'epithelial abrasion' should, in the great majority of cases, take the place of 'ulceration;' and I believe that the words 'irritation' or 'relaxation' should generally take the place which has been assigned to 'inflammation.' The changes in the uterus, and the increased secretions of the uterus and vagina, found in cases of leucorrhœa, are not such as attend inflammation in other parts of the body. It is not after an attack of acknowledged metritis that leucorrhœa is most prone to occur. The discharge generally comes on in so slow a manner that its advent cannot often be referred to any particular date. No doubt in some cases—as after suppression of the catamenia from cold or imprudence; after abortion or parturition, or mechanical injury—a genuine inflammatory state lays the foundation of leucorrhœa; but the leucorrhœal discharge and the local irritation constantly remain long after the signs of positive inflammatory disease have passed away. Chronic irritation and relaxation, rather than chronic inflammation, is the state which generally obtains under these circumstances. The most common and immediate cause of leucorrhœa is simple irritation of the glands of the cervical canal, and many of the conditions described as inflammatory, such as abrasions and indurations of the os and cervix uteri, are, as I have repeatedly observed the results of the long continued discharge, rather than of inflammation occurring in the os and cervix as a primary affection."

In the treatment of leucorrhœa, Dr. S. remarks that undue prominence must not be given to either constitutional or local medication. In some cases, constitutional measures alone will be sufficient to arrest the disease; in others, this may be effected by local means; but in the great majority of cases, both constitutional and local measures will be necessary to effect a permanent cure. The general principle of treatment, we are told, must be the arrest of the discharge, the removal of the local disorder upon which the discharge depends, and the relief of any constitutional disorder with which the leucorrhœa may be connected, either as cause or effect.

To fulfil these indications, the remedies noticed by Dr. S. are preparations of iron, a combination of iron and alum, tonics, purgatives, vaginal injections, caustic application, pessaries, cubebs and matico, etc., bathing, change of air, rest, and recumbency.

The comments of the author upon each of these remedies, and the particular circumstances and stages of the disease to which they are respectively adapted, are full and interesting. His remarks on the abuse of cauterization are particularly opportune. There are too many practitioners who, in almost every case of impaired health in the female, diagnose disease of the neck of the womb, and, as a necessary consequence, introduce the speculum and make repeated applications of the nitrate of silver to ulcers of the cervix uteri, either real or imaginary.

We thank Dr. Smith for his very excellent monograph, and very earnestly recommend it to the notice of American practitioners. No one, we are persuaded, can rise from its perusal without having acquired more definite and correct views of the pathology of leucorrhœa, and a clearer conception of its proper treatment under the several forms, and with the different complications it is liable to occur.—*American Journal Medical Sciences.*

